# A Fatal Case of Presumptive Diagnosis of Leptospirosis Involving the Central Nervous System

**DOI:** 10.3390/healthcare12050568

**Published:** 2024-02-29

**Authors:** Christina Alexopoulou, Athanasia Proklou, Sofia Kokkini, Maria Raissaki, Ioannis Konstantinou, Eumorfia Kondili

**Affiliations:** 1 Department of Intensive Care Medicine, University Hospital of Heraklion, 71500 Heraklion, Greece; proklath@gmail.com (A.P.); sofkok@hotmail.com (S.K.); konstantinou.ioannis@yahoo.gr (I.K.); kondylie@uoc.gr (E.K.); 2 Radiology Department, University Hospital of Heraklion, 71500 Heraklion, Greece; mraissaki@yahoo.gr

**Keywords:** Leptospirosis, CNS, coma, Weil’s syndrome, case report

## Abstract

Leptospirosis is a reemerging zooanthroponosis with a worldwide distribution, though it has a higher incidence in areas with tropical climate. A characteristic finding of the disease is its wide spectrum of symptoms and organ involvement, as it can appear either with very mild flu-like manifestations or with multiorgan failure, affecting the central nervous system (CNS) with a concomitant hepatorenal dysfunction (Weil’s syndrome) and significant high mortality rate. We report herein a fatal case of a 25 years old female, previously healthy, with impaired neurological status. She had high fever and severe multiorgan failure. The clinical data and the epidemiological factors were not conclusive for the diagnosis, and the first serology test from the cerebrospinal fluid (CSF) and sera samples were negative. When the repetition of the blood test showed elevated IgM antibodies, Leptospirosis was the presumptive diagnosis. Although CNS involvement is rare, the diagnosis should be considered when there is an elevated risk of exposure. The diagnostic protocol should encompass direct evidence of the bacterium and indirect measurement of antibodies. Timely detection and management are imperative to forestall complications and fatality associated with the disease.

## 1. Introduction

Leptospirosis is a reemerging zooanthroponosis caused by spirochetes of the genus *Leptospira*. Different ancient reports from the Far East had described febrile icteric illness, probably leptospirosis, as a ‘rice field jaundice’ in the rice-harvesting Chinese population, or as ‘Akiyami (autumn fever)’ in Japan. Moreover, in European texts, the disease was described as ‘cane-cutter’s disease’ or ‘swineherd’s disease’ [1]. In 1886, Adolph Weil in Heidelberg described leptospirosis as a syndrome with multiorgan manifestations, high fever, concomitant icterus, enlarged spleen, acute renal impairment, and conjunctivitis. The causative organism was demonstrated a few years later by Stimson, who identified it by silver staining the presence of clumps of spirochetes in the kidney tubules of a patient who died of yellow fever. Stimson named them *Spirochaeta interrogans*, from their hooked ends resembling a question mark [1,2].

Though the distribution of leptospirosis is worldwide, an incidence rate of more than 10 cases per 100,000 of the population is recorded in tropical climates and significantly less (0.1–1 per 100,000) in temperate climates [3]. East Sub-Saharan Africa, the Caribbean, Oceania, and South East Asia are related, with more than 70% of reported cases of leptospirosis. While the worldwide prevalence of the disease has remained stable, it tends to occur more frequently following natural disasters such as flooding or earthquakes. Extended periods of hot, dry weather can also lead to increased leptospires in freshwater ponds and rivers, making swimming, canoeing, white water rafting, fishing, and other water sports potential risk factors for outbreaks. Young individuals who work in farming and are in contact with livestock and those exposed to rodents at their workplaces, particularly males, are at a higher risk of infection [4].

Although any mammal can be an animal reservoir for the organism, small mammals are the most important maintenance hosts, as they can transfer the pathogen to domestic farm animals, dogs, and humans through urine. The transmission of leptospirosis to humans occurs by direct exposure to the urine of infected animals or urine-contaminated water and soil. This can happen through recreational or occupational activities. The contaminated water or soil infects humans via abrasions or cuts in the skin, the conjunctiva, or the gastrointestinal route. Prolonged water immersion can also lead to infection, particularly if skin abrasion occurs. In rare instances, transmission can also occur through inhalation of infected water or aerosols through the mucous membranes of the respiratory tract or following an animal bite [2]. Although human-to-human transmission is uncommon, there have been reports of transmission during sexual intercourse [5].

Leptospirosis is frequently underdiagnosed as it can manifest through a broad spectrum of nonspecific signs and symptoms that involve multiple organs. In certain geographic locations, a mild, self-limiting, acute febrile illness may be present, while in tropical and high-risk areas, a severe, life-threatening form with multiple organ failure is described. Dengue and other hemorrhagic fevers, rickettsial infection, malaria, and even common bacterial sepsis, can be confused with the initial presentation of leptospirosis. The most common presentation of the infection is the anicteric form, resembling seasonal influenza, while icterohemorrhagic leptospirosis, or Weil’s syndrome, represents the most severe form of the disease with significant morbidity and mortality of 40%. About 10% of the infected population will develop a severe form of the disease [6].

The incubation period can vary from 2–20 days, with a mean range between 7–12 days. The disease often has a biphasic clinical presentation with an acute or leptospiremic phase occurring in the first week, followed by an ‘immune phase’ [7]. During the first phase, fever, myalgias (especially in the paraspinal muscles with signs of meningism, or the abdominal muscles), chills, and headache (with retro-orbital pain and photophobia) are the main nonspecific symptoms. On the third to fourth day, conjunctival suffusion, and less often a transient skin rash, can be presented. The second phase of the disease is characterized by IgM production, blood release, and excretion of leptospires in the urine, as spirochaetes settle in the proximal tubules of the kidney. During this phase of the disease, most complications can occur depending on the degree of organ involvement and the virulence of the organism [6]. The exact pathogenetic mechanisms of the most severe form of the disease are not completely understood yet, and the most severe form of the disease is thought as a form of vasculitis. The clinical presentation includes jaundice as a prominent feature, and severe septic shock with multiple acute organ involvement, mainly kidneys, brain, and lungs. Death is often related to multiple organ failure and diffuse alveolar hemorrhaging. Leptospirosis involves the nervous system in around 10–15% of the cases, mainly in the form of aseptic meningitis. However, other atypical forms of nervous system involvement can also occur, including intracranial bleeding and thrombosis, Guillain–Barré syndrome, and ocular manifestations in the form of uveitis and optic neuritis [8]. Diagnosis of leptospirosis is challenging in many cases. It is based on suggestive clinical symptoms with a history of risk exposure. Increased clinical suspicion is crucial for the severe form of the disease. We report herein a case of a 25-year-old female who presented with Weil’s syndrome and severe brain involvement. Despite targeted therapy, the patient died from multi-organ failure.

## 2. Case Description

A 25-year-old female with an unremarkable past medical history was transferred to the emergency department of a regional insular hospital of Greece after an abrupt loss of consciousness and a fainting episode in a public place, unconscious with a Glasgow Coma Scale (GCS) 6/15, febrile (40 °C body temperature), and a macular degenerative hemorrhagic rash of the forehead and periocular areas. She was intubated, and a brain Computed Tomography (CT) scan was performed with unremarkable findings. A lumbar puncture was performed, and both cerebrospinal fluid (CSF) biochemical and microbiological tests were normal. Specifically, the CSF investigation included the following: cytology: 10 cells per field of view, 7680 red blood cells per field of view, microbiology: Gram stain was negative, as was the culture and biochemistry: glucose ratio (CSF/blood) was 55% (reference range for normal above 50%), and protein was 35 mg/dL (reference range 15–45 mg/dl); polymerase chain reaction (PCR) for common encephalitis-meningitis pathogens was negative and included the following: *Cytomegalovirus (CMV), Varicella Zoster virus (VZV), Herpes Simplex virus 1/2 (HSV1/2), Epstein–Barr virus (EBV), Herpex virus 6 (HHV6), Enterovirus, Parechovirus, Escherichia coli k1, Haemophilus influenzae, Listeria monocytogenes, Neisseria meningitidis, Streptococcus agalactiae, Streptococcus pneumoniae,* and *Cryptococcus neoformans/gattii.*

The patient was initially admitted to the Intensive Care Unit (ICU) of the insular regional hospital. One day later, she was transferred to the ICU of our University Hospital for advanced organ support due to clinical deterioration and multiple organ failure. During her admission, she appeared on substantial hemodynamic instability on norepinephrine 0.5 mcg/kg/min and tachycardia (120 rpm). At that time, aggressive fluid resuscitation, vasopressor, and empirical broad-spectrum antibiotic therapy (cefepime [Zefepime, Vocate] 2 gr twice per day and vancomycin [Voxin, Vianex] 2 gr continuous infusion) were started for presumed septic shock. Initial investigations revealed severe thrombocytopenia (8 K/μL, reference values 150–450 K/μL), elevated creatinine kinase enzyme (CPK = 5520 U/L, reference values < 172 U/L), elevated international normalized ratio (INR = 2.66, reference values 0.85–1.2) and D-dimers > 35.20 (reference values 0–0.55 mg/L), low fibrinogen levels (128.1, reference values 210–400 mg/dL), elevated liver function tests (alanine transaminase 362 U/L (reference values < 35 U/L), and aspartate aminotransferase 562 U/L (reference values < 35 U/L) and mild renal impairment.

On admission to our department, differential diagnosis included a wide range of diseases which had to be excluded. The main symptoms, laboratory tests, and results for the first eight days are summarized at Table 1. The patient presented acute liver and renal failure, severe thrombocytopenia, CNS involvement, and non-generalized rash consisted of non-palpable purpura localized around the left eye-periorbital and on the left breast accompanied with ecchymosis.

Laboratory results for autoimmune diseases were as follows: ANA 1:20 (negative, reference range < 1:20), ANCA 1:80 (negative, reference range < 1:80), RF <10.1 (negative, reference ratio 0–35 IU/mL), Anti B2-GPI IgM 0.36 (negative, reference range < 12 U/mL), Anti B2-GPI IgG 1.61 U/mL, reference range < 12 U/mL), C4: 15 mg/dL (reference range 15–47 mg/dL), C3: 88 mg/dL (reference range 87–187 mg/dL), IgM: 74,60 mg/dL (reference range 25–170 mg/dL), IgG: 765 mg/dL (reference range 701–1600 mg/dL), and IgA: 151 mg/dL (reference range 48–368 mg/dL). Autoimmune diseases, mainly systemic lupus erythematosus (SLE) vasculitis, were excluded based on negative laboratory results (AΝA, ANCA, etc.) and on the absence of arthritis, generalized rash, and other diagnostic criteria. Purpura was attributed to the severe underlying thrombocytopenia. The urine sediment findings were consistent with acute kidney injury without any signs of glomerulonephritis or interstitial nephritis, and were attributed to the substantial hemodynamic instability on presentation and possibly during the transfer of the patient. The low complement levels (54.7 mg/dL, reference values 87–187 mg/dL), as well as the high levels of ferritin (5066 ng/mL, reference values 4.63–204 ng/mL), were attributed to acute liver failure, and were restored in the next days of hospitalization. Veno-occlusive disease was excluded by an abdomen computed tomography angiography (CTA).

A full-body CT scan revealed unremarkable findings, except for ground glass with concomitant consolidation mainly on the left lung and to a lesser extent on the right lower lobe. Abdominal organs presented normal on the CT scan without hepatomegaly or splenomegaly. All CT scans were performed on a 128-slice CT scanner (Revolution GSI; GE Healthcare, Chicago, IL, USA).

However, no schistocytes were revealed on peripheral blood smear. The blood, urine and bronchial specimen’s cultures revealed no pathogen. The blood culture system used was BacT/ALERT, in bottle types BacT/ALERT FA PLUS and BacT/ALERT FN PLUS containing aerobic and anaerobic media, respectively, with adsorbent polymeric resin beads. The method used for monitoring growth was colorimetric change caused by a drop in pH from increased CO_2_ levels. Urine cultures were inoculated in Drigalski Agar, Columbia ANC agar +5% sheep blood and Trypticase™ Soy Agar, BD Diagnostics. Bronchial specimens were inoculated in Columbia agar +5% sheep blood and Chocolate Agar with Vitox.

A broad serological testing was carried out including *CMV, Hepatitis A virus (HAV), VZV, Adenovirus, Parvovirus, Echovirus, Coxsackie, HSV1/2, Measles, Hepatitis C virus (HCV), Hepatitis B virus (HBV), Human Immunodeficiency virus (HIV), EBV Rickettsia typhi, Rickettsia conorii, Trichinella, Cryptococcus species, Brucella (Wright/Rose Bengal), Treponema (PRP), Salmonella (WIDAL), Borellia burgdorferi, Bordetella pertussis, Plasmodium, Leishmania,* and *Leptospira.* All results were negative.

Based on hematologist consultation, a bone marrow biopsy was performed to exclude acute hemophagocytic syndrome. The peripheral blood smear revealed 88% neutrophils, 5% lypmphocytes, and 6% monocytes with a total number of 6.400 K/μL WBCs. Schistocytes, two per field of view and two erythroblasts per field of view were also present. Phagocytosis in bone marrow was present in the bone marrow smear. The bone marrow biopsy showed hematopoietic marrow normal, in relation to age and cellularity (the hematopoietic cellular elements accounted for about 50–55% of the area of myelochores). All three hematopoietic lines were recognized with a ratio of medullary to rubella 2/1. Normal maturation of medullary. Normal number of megakaryocytes without appreciable dysplasia. Immature CD34+ cellular elements corresponding to less than 1% of the marrow, without obvious fibrosis. Immunohistochemicals (PGM1, CD16) showed increased number of mast cells in the cytoplasm, in some of which nucleated rubella were observed. There was mild swelling of the interstitial web.

Treatment with corticosteroids and Immunoglobulin G (Octagam 10%, Octapharma) as rescue therapy and empirical antibiotic therapy with cefepime (Zefepime, Vocate) and vancomycin (Voxin, Vianex) for bacterial meningitis was started. Two days later, a new lumbar puncture was performed. CSF showed leukocytes at 10 cells/mm^3^, glucose at 95 mg/dL (reference values 40–70 mg/dL) with a ratio of 58% (normal value above 50%), and protein at 109 mg/dL (reference values 15–45 mg/dL). Both the CSF culture and PCR were negative for bacterial or viral pathogens, including *CMV, VZV, HSV1/2, EBV, HHV, Enterovirus, Parechovirus, Escherichia coli k1, Haemophilus influenzae, Listeria monocytogenes, Neisseria meningitidis, Streptococcus agalactiae, Streptococcus pneumoniae,* and *Cryptococcus neoformans/gattii.*

**Figure 1:**
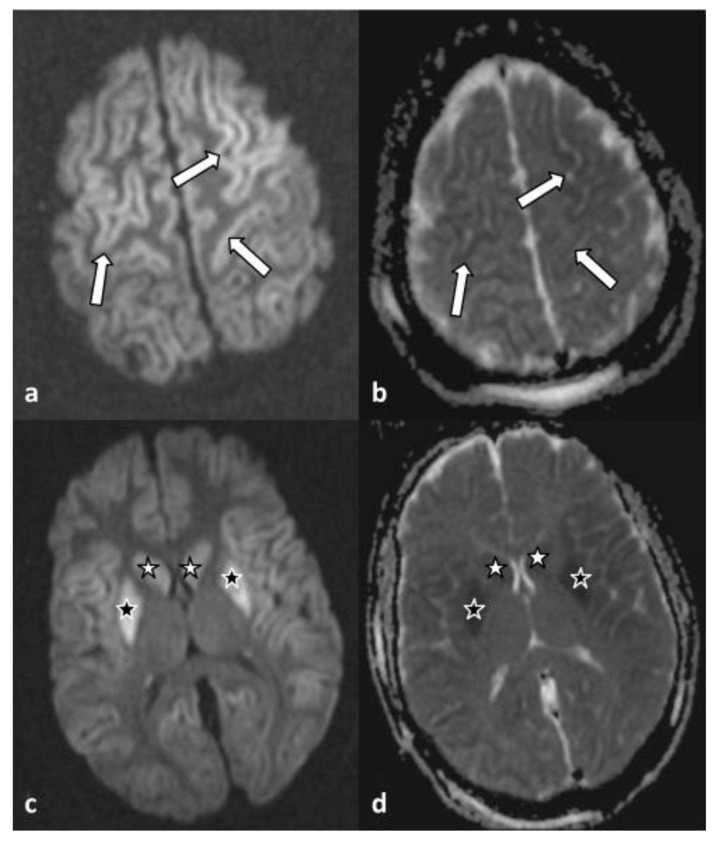
Axial diffusion-weighted imaging (**a**) and apparent diffusion coefficient map (**b**) at the level of the convexity, showing increased and decreased signal intensity of the cerebral cortex along both frontal lobes (arrows), respectively. Axial diffusion-weighted imaging (**c**) at the level of basal ganglia reveals symmetrical hyperintensity of the caudate (white asterisk) and lentiform (black asterisk) nuclei, bilaterally. On the corresponding apparent diffusion coefficient map (**d**), the same areas appear hypointense.

A habit history of frequent spring water consumption reported by her closest contact, along with the reports of endemic tropical diseases on the island of her residency, raised the suspicion of leptospirosis infection, although the serological test of the first sample was negative. Based on that, eight days later, a second blood sample revealed positive IgM against *Leptospira* (immunochromatographic assay) and confirmed the diagnosis. Targeted therapy with 2 g of ceftriaxone intravenously, which was started empirically on the second day based on high grade of clinical suspicion was completed. On the 5th day, an attempt of analgosedation discontinuation was interrupted as the patient presented multiple episodes of myoclonus, exophoria, and impaired level of consciousness (GCS 6/15). A brain magnetic resonance imaging (MRI) revealed findings indicative of cytotoxic edema in the context of global hypoxic ischemic injury (Figure 1) of the caudate (white asterisk) and the lentiform (black asterisk) nuclei, bilaterally. On the corresponding apparent diffusion coefficient map (d), the same areas appeared hypointense.

**Figure 2:**
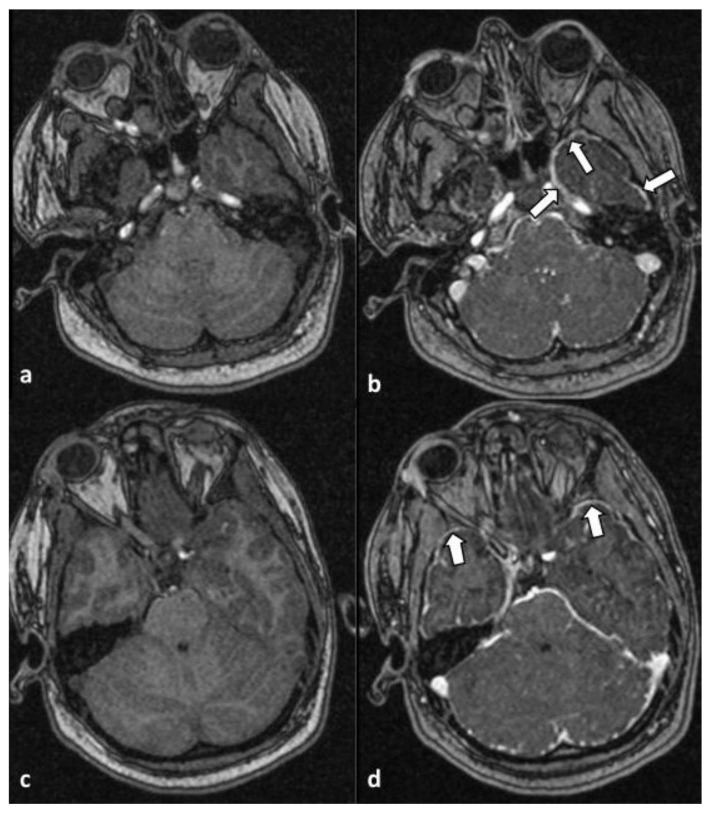
Axial T1-weighted MR images without (**a**,**c**) and after (**b**,**d**) administration of intravenous contrast material. An infratentorial leptomeningeal enhancement was noted, most prevalent along the anterior aspect of the temporal lobes (arrows). The findings were suggestive of the presence of meningitis/meningoencephalitis.

Moreover, infratentorial leptomeningeal enhancement, most prevalent along the anterior aspect of the temporal lobes (arrows), was observed in axial T1-weighted MR images without (a and c) and after (b and d) administration of intravenous contrast. The findings were suggestive of the presence of meningitis/meningoencephalitis (Figure 2).

Despite targeted antibiotic therapy and multiple organ advanced supportive therapy, she presented no neurological improvement, remaining on a low level of consciousness and ventilator dependency. Furthermore, her hospitalization was complicated by multiple episodes of multidrug-resistant bloodstream infections, and ventilation-associated pneumonia, and ultimately the patients died two months later. Bloodstream infections were attributed to catheter-related infections and ventilator-associated pneumonias. The identified pathogens were *Pseudomonas aeruginosa* multidrug-resistant (MDR) and *Acinetobacter baumannii* MDR, as confirmed by positive blood cultures and bronchoalveolar lavage specimens. Further analysis revealed *Pseudomonas aeruginosa* resistance to β-lactams, quinolones, and carbapenems, while it exhibited sensitivity to colistin and aztreonam. *Acinetobacter baumannii*, on the other hand, demonstrated resistance to carbapenems, colistin, aminoglycosides, and ampicillin-sulbactam, with sensitivity noted to minocycline and intermediate sensitivity to tigecycline (MIC: 4 μg/mL). In response to these findings, targeted antibiotic therapy was administered based on antibiograms (colimycine Colistin Norma, tigecycline Tygacil Anfarm, ceftazidime Septax Vianex). Unfortunately, despite these efforts, the patient succumbed to multiorgan failure, attributed to multiple episodes of multidrug-resistant bloodstream infections leading to sepsis. The underlying cause of these recurring infections is believed to be associated with the patient’s prolonged stay in the ICU, driven by ventilator dependency and a diminished level of consciousness following the initial insult of leptospirosis.

## 3. Discussion

Leptospirosis is a significant global health concern, substantiated by a 2017 Chinese study that documented a case-fatality ratio of 26.89 out of 7587 cases over a decade. Furthermore, Costa et al. approximated that there are 1.03 million cases of leptospirosis each year worldwide, contributing to an estimated 58,900 deaths. The authors also observed that the mortality rate of leptospirosis is equivalent to, or even surpasses, that of hemorrhagic fever in low-income and tropical countries. They concluded that leptospirosis is a primary cause of morbidity and mortality among zooanthroponotic diseases [4,9].

According to the Greek National Public Health Organization (GNPHO), in Greece, leptospirosis is a rare bacterial disease that affects approximately 20 individuals each year, resulting in an incidence rate of 0.13 to 0.31 per 100,000 population. However, the incidence rate may differ depending on the geographic region and season. It is important to note that Leptospirosis is likely underdiagnosed and underreported, which suggests that the actual incidence rate may be higher than the reported rate [10]. Recent studies conducted in southwestern Greece have estimated the incidence rate of Leptospirosis to be approximately 1.1 cases per 100,000 population in the area. The increased incidence rate may be attributed to climate change and the agricultural practices of the local population [11].

The clinical manifestation of the disease varies broadly from mild non-specific symptoms to multiple organ failure. Although the severe form of Weil’s disease is rare, it can lead to life-threatening complications. The most common CNS manifestation is aseptic meningitis, accounting for 5% to 13% of all cases. Nevertheless, myelopathy, Guillain-Barre syndrome, meningoencephalitis, cerebellitis, cerebral hemorrhage, tremor neuralgia, facial palsy mononeuritis, movement disorders, and hemiplegia may also occur. Importantly, the clinical course of CNS in Weil’s syndrome is usually associated with reversible neurological status [8]. The underestimation of leptospirosis mortality and morbidity rates in various countries is a topic of significant concern. Hartskeerl et al., have highlighted the absence of notification and epidemiological efforts as the primary reasons for this underestimation. Furthermore, the incidence rate of the disease is estimated only among individuals with severe leptospirosis around the world, leaving out those with milder cases. This exclusionary approach has resulted in a distorted perception of the actual incidence of the disease globally. The true extent of the impact of leptospirosis must be accurately documented and reported so that appropriate measures can be taken to address this health concern [12].

We present herein a case of Weil’s syndrome associated with irreversible CNS disease. The presenting sign of the patient was coma, and she remained on a low conscious level over the clinical course. The combination of severe thrombocytopenia, altered mental status, and acute kidney injury raised the suspicion of possible thrombotic thrombopenic purpura (TTP), but the absence of schistocytes on peripheral blood smear excluded the diagnosis. The diagnosis of an infection, causing multiorgan failure attributed to disseminated intravascular coagulation (DIC) in combination with hypoperfusion seemed to be the most possible diagnosis. A thorough diagnostic process ruled out the most potential causes of coma, including common causes of bacterial meningitis, acute cerebrovascular accident, and thrombotic TTP with cerebral involvement. The positive serological test, combined with clinical data and environmental factors and the exclusion of other causes of coma, reinforced the diagnosis of leptospirosis with CNS involvement.

Coma as a presenting symptom in Weill’s disease is uncommon. Only 10–15% of the patients may have a neurological symptom which often remains unrecognized [12,13]. In a small cohort of patients with leptospirosis with CNS involvement, altered sensorium and seizures have been reported as the most common neurological symptoms [14]. Two retrospective studies investigated the clinical characteristics in critically ill patients with severe leptospirosis. Samrot et al. reviewed 55 leptospirosis cases admitted to the ICU during 1998–2018 in tropical Australia. They reported no significant neurological signs except headache in 71% of hospital admissions [15]. Delmas et al. reported that a 4% of 134 consecutive leptospirosis cases presented with CNS involvement in the form of meningitis and encephalitis on admission. The mortality rate (identical for ICU, hospital mortality, and 28 d mortality) was 6.0% (95% CI, 2.6–11.4, eight patients) [16]. There are also a few case reports in the literature with CNS manifestation as a primary symptom of leptospirosis. All the patients suffered from headache, some of them also had symptoms such as vomiting, fever, and chills [14,17,18,19,20]. One patient had sudden onset of paraparesis, two had seizures, and one patient appeared with decreased responsiveness to commands, decreased verbal output, and inability to move his limbs [14,20,21,22]. Bandara et al. present two cases of neuroleptospirosis with headache, vomiting, and photophobia [23], and Zhang et al. reported a case of Leptospirosis-associated meningitis in a patient with sjögren’s syndrome [24].

In a recent brief report from India, the authors analyzed seven consecutive patients with neuroleptospirosis admitted to a neurology ward. They had aseptic meningitis due to *Leptospira* without any systemic manifestation and they all recovered after antibiotic therapy with ceftriaxone and doxycycline [25].

In a study conducted in southwestern Greece among 45 patients with leptospirosis, only one patient presented CNS manifestations, specifically aseptic meningitis [11]. Neurological manifestations of leptospirosis are rare, and the most common is aseptic meningitis. The only report that we found in the literature that is similar to our case dates back to 2002 and involves a patient who presented with fever, coma, petechial rash, and conjunctival suffusion. The patient also had low PLTs and altered liver function. Leptospirosis was suspected, and penicillin and doxycycline were administered on day two despite the absence of laboratory evidence of leptospirosis. Serum IgM antibodies against *Leptospira* were negative, while IgG antibodies were positive. A four-fold increase in the titer of IgG antibodies confirmed the diagnosis one week later. After an extended stay in the ICU, the patient eventually passed away from a bloodstream infection [26]. In our case, our patient had negative IgM and IgG antibodies on the first evaluation upon admission, but after eight days the IgM antibodies were positive, confirming the diagnosis eventually. Although we started ceftriaxone on day two due to clinical suspicion, the patient died after two months in the ICU due to multiple episodes with multidrug resistant bloodstream infections and ventilator-associated pneumonia without any recovery from brain damage.

Although neuroleptospirosis is rare and coma even rarer, a history of risk exposure with symptoms such as headache, myalgia, jaundice, aseptic meningitis, and multiorgan failure will lead to the suspicion of leptospirosis, and appropriate diagnostic tests could confirm the diagnosis.

Diagnostic procedure includes direct methods of identifying the bacterium and indirect methods such as serological tests. Blood culture is the gold standard and has to be performed during the first week of the illness. Unfortunately, this is a demanding method with low diagnostic capability, useful to determine antibiotic sensitivity. PCR has to be performed in the first week, and shows high sensitivity and specificity. Serological methods detect antibodies by day six to ten of illness, and during their peak within three to four weeks. It is useful to measure antibodies with an interval of two weeks to compare them. This comparison has a high sensitivity and specificity [6].

In our case, the confounding factors of habitual spring water consumption, along with the reports of endemic tropical diseases on the island of her residency, raised the suspicion of leptospirosis infection, although the first serology was negative. Based on that, eight days later, a second blood sample revealed positive IgM against *Leptospira* (immunochromatographic assay) and confirmed the diagnosis.

The methodology used for the diagnosis of *Leptospira* was the GenBio IgM ImmunoDOT *Leptospira* test, which is a qualitative enzyme immunoassay that specifically detects IgM antibodies to *Leptospira biflexa* (serovar patoc 1). The test is intended for use in serum, plasma, heparinized whole blood, or finger-stick capillary blood. The microscopic agglutination test (MAT) is the preferred method, and is the current World Health Organization standard reference method [27]. The MAT method has high sensitivity and specificity, and permits the detection of group-specific antibodies. Despite its widespread application, the MAT method is constrained to highly specialized laboratories capable of maintaining live, hazardous stock serovar cultures. Criteria for laboratory diagnosis, as outlined by the Centers for Disease Control and Prevention (CDC), involve the isolation of leptospira from a clinical specimen, a significant four-fold or greater increase in *Leptospira* agglutination titer between acute and convalescent-phase specimens collected at intervals of greater than or equal to two weeks, or the visualization of *Leptospira* in a clinical specimen by immunofluorescence [28].

Presumptive diagnostic criteria are based on a leptospira agglutination titer equal to or exceeding 200 in single specimens from clinically symptomatic cases. Alternative methods for the detection of IgM antibodies during the acute phase, such as enzyme-linked microplate immunosorbent (ELISA) assays and an indirect hemagglutination (IHA) in vitro diagnostic procedure, are commercially available and reported to exhibit sensitivity and specificity comparable to the MAT method [29,30,31]. It is noteworthy that the MAT method has not been commercially distributed.

According to modified Faine’s criteria for diagnosis of leptospirosis, our patient had a score of >25 (Part A, B, C/rapid test), which means that the diagnosis of leptospirosis is presumptive, with a sensitivity 89.39% and specificity of 58.82%, while the parts A + B + C/MAT ≥ 1800 and/or PCR had a sensitivity of 98.31% and specificity of 55.05 [30,31].

## 4. Conclusions

Leptospirosis remains a reemerging zooanthroponosis. Although CNS involvement does not appear to be the main presenting symptom, legionella still must be excluded in cases of aseptic meningitis with compatible epidemiological background. A high index of suspicion and a careful history of exposure is essential in order to consider leptospirosis as a possible diagnosis and start empirical treatment without waiting for the laboratory confirmation.

## Figures and Tables

**Table 1:** Main symptoms, laboratory tests and results during the first 8 days.

Day 0 Hospital in rural area	Coma, fever (40 °C), meningism, and a macular degenerative hemorrhagic rash of the forehead and periocular areas Respiratory failure	Brain CT scan Lumbar puncture	Normal
		PCR of CSF for common pathogens	Negative
Day 1 Our hospital	Transfer to our hospital Severe shock Acute liver and renal failure, severe thrombocytopenia, non-palpable purpura localized around the left eye-periorbital and on the left breast accompanied with ecchymosis.	Cultures: Blood, Bronchial specimen, urine Broad serological tests Blood ImmunoDOT *Leptospira* test	All negative
		ANA ANCA Anti B2-GPI IgM Anti B2-GPI IgG C3, C4, IgM, IgG, IgA	All normal
Day 2	Intubated Hemodynamically stable High clinical suspicion of legionella	CT angiography of brain abdomen Lumbar puncture Bone marrow biopsy	All normal
Day 5	Analgosedation discontinuation Myoclonus Exophoria Coma	Brain MRI	Cytotoxic edema in the context of global hypoxic-ischemic injury
Day 8	No improvement	Blood ImmunoDOT *Leptospira* test	Elevated IgM

## Data Availability

Data are contained within the article.
